# Cross-Sectional Study on Zoonotic Bacteria Carriage by Small Ruminants from Portugal’s Central Region

**DOI:** 10.3390/pathogens14111081

**Published:** 2025-10-23

**Authors:** Maria Aires Pereira, Alexandra Lameira Baptista, Rita Cruz, Fernando Esteves, Ana Amaro, João R. Mesquita, Elizabete Almeida, Joana Braguez, Madalena Malva, Alda F. A. Pires

**Affiliations:** 1 Instituto Politécnico de Viseu, Escola Superior Agrária de Viseu, Campus Politécnico, 3504-510 Viseu, Portugal; alexabaptista@esav.ipv.pt (A.L.B.); rcruz@esav.ipv.pt (R.C.); festeves@esav.ipv.pt (F.E.); 2 CERNAS-IPV Research Centre, Instituto Politécnico de Viseu, Campus Politécnico, Repeses, 3504-510 Viseu, Portugal; 3 Global Health and Tropical Medicine, GHTM, Associate Laboratory in Translation and Innovation Towards Global Health, LA-REAL, Instituto de Higiene e Medicina Tropical, IHMT, Universidade NOVA de Lisboa, UNL, Rua da Junqueira 100, 1349-008 Lisboa, Portugal; 4 Universidade de Trás-os-Montes e Alto Douro, Quinta de Prados, 5000-801 Vila Real, Portugal; elizabete.catarina@alsglobal.com; 5 Epidemiology Research Unit (EPIUnit), Instituto de Saúde Pública, Universidade do Porto, 4050-091 Porto, Portugal; jrmesquita@icbas.up.pt; 6 Laboratory for Integrative and Translational Research in Population Health (ITR), 4050-600 Porto, Portugal; 7 Laboratory of Bacteriology and Mycology, National Reference Laboratory of Animal Health, INIAV—National Institute of Agrarian and Veterinary Research, 2780-157 Oeiras, Portugal; ana.amaro@iniav.pt; 8 School of Medicine and Biomedical Sciences (ICBAS), University of Porto, 4050-313 Porto, Portugal; 9 Australian Laboratory Services (ALS), Zona Industrial de Tondela, ZIM II Lote 2 e Lote 6, 3460-070 Tondela, Portugal; 10 Instituto Politécnico de Viseu, Escola Superior Tecnologia e Gestão, Av. Cidade Politécnica, 3504-510 Viseu, Portugal; jbraguez@estgv.ipv.pt (J.B.); malva@estgv.pt (M.M.); 11 CEIS20—Centro de Estudos Interdisciplinares, Rua Filipe Simões n° 33, 3000-186 Coimbra, Portugal; 12 Department of Population Health and Reproduction, School of Veterinary Medicine, University of California, Davis, CA 95616, USA; apires@ucdavis.edu

**Keywords:** antimicrobial resistance, sheep, goat, *Campylobacter* spp., *Salmonella* spp., extended spectrum β-lactamase-producing strains of *Escherichia coli*, methicillin-resistant *Staphylococcus aureus*

## Abstract

Zoonotic bacteria, namely *Campylobacter* spp., *Escherichia coli*, *Salmonella* spp. and *Staphylococcus aureus*, are commonly implicated in human infections and can be transmitted from animals to humans through direct contact, the environment or the food chain. The emergence of antimicrobial resistance in these zoonotic bacteria, namely extended spectrum β-lactamases (ESBLs)-producing strains of *E. coli* and methicillin-resistant *S. aureus* (MRSA), has become a public health concern worldwide. This study aimed to determine the prevalence of fecal carriage of *Campylobacter* spp., *Salmonella* spp. and ESBL-producing strains of *E. coli*, as well as nasal carriage of MRSA, and to identify risk factors associated with the presence of these zoonotic bacteria in small ruminants from Portugal’s Central Region. A total of 732 animals, of which 432 sheep and 300 goats from 122 farms were sampled. Zoonotic enteric bacteria were isolated from individual fecal samples, while MRSA were isolated from pooled nasal swabs collected from each farm. Bacteria were isolated according to standard microbiological methods. The overall prevalence of *Campylobacter* spp. and *Salmonella* spp. at the animal level was 15.6% and 8.3%, respectively, with significantly higher prevalence in sheep (19.0% and 12.7%) compared to goat (10.7% and 2.0%). Presumptive ESBL-producing strains of *E. coli* was isolated from 5.2% of the animals. Presumptive MRSA was isolated from 5.7% of the farms. A cluster analysis was performed to identify farm clusters with similar characteristics based on the isolation of *Campylobacter* spp., *Salmonella* spp., presumptive ESBL-producing *E. coli*, and presumptive MRSA. Farms were grouped into three clusters: “Resistant”, “Campylobacter” and “Salmonella”. The likelihood of farms belonging to “Campylobacter” and “Salmonella” clusters in comparison to “Resistant” cluster was associated with animal species, farm location, and farmer’ practices regarding antibiotic use. This study reinforces the role of small ruminants as asymptomatic reservoirs of *Campylobacter* spp., *Salmonella* spp., *E. coli* and *S. aureus* and confirms, for the first time, their role as carriers of presumptive antibiotic-resistant zoonotic bacteria in Portugal’s Central Region.

## 1. Introduction

Small ruminant production plays an important role in the sustainability of underserved rural regions. It represents a viable economic activity for the local populations and contributes to the maintenance of rural communities by helping prevent the desertification of these areas. Sheep and goats, particularly autochthone breeds, are well adapted to rough terrain and have a greater capacity to utilize available nutrients where forage productivity is low. Additionally, extensive and semi-extensive production systems contribute to help prevent forest fires and soil erosion [1].

In Portugal, small ruminants are primarily raised for their meat and milk. Lambs and kids from dairy and meat farms are sold for human consumption, and milk from dairy farms is primarily used for cheese production. The economic valorization of traditional products (i.e., meat and cheese) of recognized quality, qualified as Protected Designation of Origin (PDO) and Indication Protected Geographical (IGP), obtained from the sheep and goats is a strategy for developing of rural areas [1]. Lamb and goat meat is highly valued by the Portuguese population and are mainly consumed during festive periods. The Serra da Estrela, Beira Baixa and Rabaçal PDO cheeses, produced with raw milk, are considered as the main representative products of Portugal’s Central Region, with qualities and characteristics that refer directly to this specific geographical area [2].

However, foodborne bacterial infections associated with the consumption of meat from small ruminants and PDO cheeses produced in this region can pose significant public health risks [3]. Indeed, a total of 49 foodborne disease outbreaks caused by small ruminant dairy products were registered in 2017 in Europe, mainly associated with *Campylobacter* spp. (40.0%), *Salmonella* spp. (20.0%), bacterial toxins (18.0%) and Shiga toxin-producing *Escherichia coli* (10%) [4]. Milk and milk products cause about 1–5% of the total of foodborne disease outbreaks [5,6]. Generally, soft cheeses and those made from raw milk are more likely to be involved in cheese-associated outbreaks [4,6].

*Campylobacter* spp., particularly *Campylobacter jejuni* and, to a lesser extent, *Campylobacter coli*, are leading causes of bacterial foodborne gastroenteritis in humans worldwide [7,8]. In ruminants, *Campylobacter* species are generally considered commensals of the gastrointestinal tract and gallbladder [8]. However, *Campylobacter fetus* subsp. *fetus* and *C. jejuni* are important causes of infectious abortion in sheep (epizootic abortion) and of sporadic abortion in cattle and goats, usually during the last trimester of gestation [8]. *Campylobacter* spp. are highly prevalent in ruminants worldwide, and there is increasing evidence of ruminants’ significant contribution to human campylobacteriosis [9,10].

*Salmonella* spp. remains one of the leading causes of foodborne illness, particularly in the European Union [11,12]. Outbreaks of salmonellosis in humans are usually caused by consuming contaminated food, particularly undercooked or raw eggs, poultry, meat, and unpasteurized dairy products. However, direct contact with infected animals or their environment can also lead to infection in humans [13,14]. *Salmonella* spp. has been found in the mesenteric lymph nodes and/or gastrointestinal tract contents of apparently healthy and diarrheic sheep and goats [15,16,17]. Some human salmonellosis outbreaks have been linked to the consumption of sheep and goats’ meat [18].

*Staphylococcus aureus* is part of the commensal flora of the skin and mucous membranes, as well as an opportunistic pathogen responsible for various diseases in humans and animals [19]. *S. aureus* is recognized as a major pathogen causing life-threatening infections of bloodstream, skin, and soft tissue in humans, as well as mastitis in lactating cows and small ruminants [19,20].

The emergence of potentially zoonotic resistant bacteria that can be transfer from animals to humans is a great concern. *Escherichia coli* is a very diverse bacteria that can range from commensal intestinal to pathogenic intestinal and extraintestinal pathogen. Intestinal pathogenic *E. coli* causes diarrhea, mostly in neonatal food-producing animals and humans [21,22]. Extended spectrum β-lactamase (ESBL)-producing strains of *E. coli* are clinically relevant in veterinary medicine since they confer resistance to penicillins, aminopenicillins, and third and fourth-generation cephalosporins (ceftiofur, cefovecin and cefquinome), which are approved for use in animals in Europe [22]. ESBL genes have been widely detected in the digestive tract of food-producing animals, including in pathogenic *E. coli* recovered from diarrheic young animals and in commensal intestinal *E. coli* [22,23]. However, there are fewer reports of ESBL-producing strains of *E. coli* in sheep and goats than in other livestock species worldwide [24,25,26,27]. Currently, ESBL-producing *E. coli* are considered a major indicator of the burden of antimicrobial resistance in animals [22]. This means that monitoring the spread of these strains can provide an insight into the prevalence of antimicrobial resistance in each geographic area [22,28].

*S. aureus* can quickly acquire resistance to antibiotics, resulting in the emergence of resistant strains that proliferate and spreading of bacteria with a broad spectrum of resistance that can survive in different environments [19]. Methicillin-resistant *S. aureus* (MRSA) has become a worldwide public health concern. The mechanism of methicillin resistance is primarily mediated by the *mecA* gene, which encodes a new penicillin-binding protein (PBP2a) with low affinity for methicillin and other b-lactams. PBP2a blocks the arrival of the antibiotics to its target site, producing resistance. The gene *mecA* is found in the genetic *loci* staphylococcal cassette chromosome *mec* (SCC *mec*) [19,29,30].

It is currently evidenced that antimicrobial resistance (AMR) is a complex, and multifactorial problem, it seems that misuse and overuse of antimicrobials in livestock appear to contribute to the emergence of resistant bacteria in humans [31]. Indeed, it has been demonstrated that food-producing animals are reservoirs of resistant commensal and pathogenic strains of some zoonotic bacteria that can be transferred to humans through direct contact, contact with contaminated environment or the food chain [32].

Considerable pressure has been placed on veterinarians to restrict antimicrobial use (AMU). New European regulations on veterinary medicines and medicated feed entered into force in 2022 and substantially influenced antimicrobial prescribing and usage throughout Europe. However, farmers must also be involved in the joint effort to reduce AMU in livestock through good animal husbandry, disease prevention, and biosafety measures [33]. It is important to understand farmers’ attitudes and practices regarding antibiotic use and its impact on AMR when planning and implementing strategies to reduce AMU [34].

The present study aimed to investigate the fecal carriage of *Campylobacter* spp., *Salmonella* spp. and ESBL-producing strains of *E. coli* and the nasal carriage of MRSA, and the factors associated with the prevalence of these zoonotic bacteria in small ruminants from Portugal’s Central Region.

## 2. Materials and Methods

### 2.1. Study Area

This cross-sectional study was carried out in the districts of Viseu, Guarda, Coimbra, Castelo Branco, and Leiria, which are located in the Central Region of the Territorial Unit for Statistical Purposes Level II (NUTS II) [35]. These five districts occupy 24,890 km^2^ that corresponds to 28.0% of the national territory. The regions have a rugged terrain and are crossed by the country’s main mountain range, which culminates in the Serra da Estrela (1991 m). The region contains the hydrographic basins of several important Iberian and national rivers (Tejo, Douro, Mondego, Vouga, Dão, etc.) [36]. The small ruminant population in Portugal’s Central Region comprises 457,870 animals distributed across 10,935 herds [37]. The average number of sheep and goats per farm is 31.4 and 9.3, respectively [38].

### 2.2. Sample Size

The sample size was calculated for a known population of 457,870 animals [37], assuming a 95% confidence level and an expected seroprevalence of 50% (the most conservative estimate). Using the formula for a finite population,

$$n=\frac{N\;Z^{2}p\left(1-p\right)}{d^{2}\;\left(N-1\right)+Z^{2}p\left(1-p\right)}$$

where *N* is the population size, *Z* is the Z-value for the desired confidence level (1.96 for 95%), *p* is the expected prevalence, and *d* is the maximum absolute error, sample sizes of 432 sheep and 300 goats were determined, corresponding to maximum errors of 4.71% for sheep and 5.66% for goats.

### 2.3. Herd Selection and Sample Collection

Herds were enrolled in this cross-sectional study between 29 April 2024, and 13 March 2025. To ensure that the sample was sufficiently heterogeneous, small ruminant farms from the five districts of Portugal’s Central Region (Viseu, Guarda, Coimbra, Castelo Branco, and Leiria) were enrolled and at each farm six animals with ages greater than 6 months old were sampled.

Herd selection was based on the following inclusion criteria: (1) herds with goat or sheep for meat or milk production, (2) herds with six or more animals, (3) located in Portugal’s Central Region, (4) having a private or official veterinarian providing veterinary services, and (5) willingness to participate. Farm selection was carried out for convenience. Its recruitment occurred through personal communications from the official or the private veterinarians. Farms were visited once by an animal science professional and a veterinarian. The majority of the farms (60.7%) were visited specifically to collect biological samples and metadata for this study. Sample collection at the other 48 farms was carried out during official health campaigns to control and eradicate diseases.

A total of 732 apparently healthy small ruminants (432 sheep and 300 goats) were randomly selected. Individual fecal samples, up to 52 g of feces, were collected directly per *rectum* using gloves and stored in labeled sterile capped containers. Rectal swabs were collected from animals without solid or semi-solid feces in the rectal ampulla. Swabs were transported in sterile medium (Deltalab, S. L., Barcelona, Spain). Nasal swab samples were taken from all selected animals. Sterile cotton swabs were inserted into the nares of both left and right nostrils and were softly rolled against the inner walls. One swab was used to collect a sample from both nostrils and was stored in liquid AMIES medium (Deltalab, S. L., Barcelona, Spain). One pool per farm was obtained, consisting of nasal swabs from six animals. After collecting, the samples were kept at 4 °C and transported to the laboratory in 24 h.

### 2.4. Questionnaire

A questionnaire was created and validated to collect metadata on the following: sociodemographic; animal signalment; production purpose (meat, milk, other), type (conventional, biologic/organic), and system, which was characterized as follows: extensive (animals permanently on pasture), semi-extensive (animals spend more time on pasture than in the barn), intensive (animals permanently housed) and semi-intensive (animals spend more time in the barn than on pasture).

Antimicrobial use (AMU) on the farm; biosafety practices; and the quality of farmer’s knowledge, attitudes and practices regarding AMU were also investigated. To assess the biosafety practices implemented on the farms one section of 17 dichotomous questions was created. The quality of farmer’s knowledge, attitude and practices regarding AMU was investigated in three sections of seven multiple-choice questions. The implementation of biosafety practices by the farmer was scored 1, while the absence of implementation was scored 0. Responses reflecting good knowledge, attitudes and practices were scored 1 point, and those reflecting poor knowledge, attitudes and practices were scored zero. The sum of the points allowed us to define the level of biosafety (scale 1–17), and the quality of the producers’ knowledge, practices and attitudes (scale 1–7 points) (Supplementary Table S1—questionnaire answers). The questionnaire was administered in-person on the day of animal sampling.

### 2.5. Microbiologycal Assays

Fecal samples were screened for the presence of presumptive ESBL-producing strains of *E. coli*, *Salmonella* spp. and *Campylobacter* spp.

ESBL-producing stains of *E. coli* isolation was performed according to EURL-AR protocol [39]. Briefly, fecal samples (1 g ± 0.1 g) were pre-enriched in 9 mL of Buffered Peptone Water (BPW) (Condalab, Madrid, Spain) at 37 °C ± 1 °C for 18–22 h. After the incubation period, the pre-enrichment broth was cultured on MacConkey Agar (Biokar Diagnostics, Paris, France) supplemented with 1 mg/L of cefotaxime at 44 ± 0.5 °C for 18–22 h. Suspect colonies (purple colonies) were subcultured on MacConkey Agar supplemented with 1 mg/L cefotaxime. Up to three colonies were subcultured individually at 37 °C ± 1 °C for 18–22 h. One of the three re-cultured colonies was selected for identification by Matrix-Assisted Laser Desorption/Ionization Time-of-Flight Mass Spectrometry (MALDI-TOF) (MALDI Biotyper^®^ Sirius–Bruker, Billerica, MA, USA). In case of positive *E. coli* identification, the colony was re-cultured to avoid contamination, and its growth was confirmed on MacConkey Agar supplemented with 1 mg/L of cefotaxime at 37 °C ± 1 °C for 18–22 h. As amplification of the resistance genes was not performed, the designation presumptive ESBL-producing stains of *E. coli* was used.

Isolation of *Salmonella* spp. was performed according to ISO 6579-1/2017 protocol [40]. Fecal samples were pre-enriched in BPW at 37 ± 1 °C for 18 ± 2 h. From this non-selective pre-enrichment broth, a 0.1 mL aliquot was transferred to 9 mL of the selective enrichment medium Modified Semi-Solid Rappaport-Vassiliadis Agar (MSRV) (CondaLab, Madrid, Spain) and incubated at 41 ± 1 °C for 24 ± 3 h. Selective plating was then performed onto xylose-lysine-desoxycholate agar (Biokar, Beauvais, France) plates and onto RAPID’Salmonella Medium (BioRad, Marnes-la-Coquette, France), incubated at 37 ± 1 °C and examined after 24 ± 3 h. Colonies of presumptive *Salmonella* were confirmed by MALDI-TOF.

*Campylobacter* spp. isolation was performed according to ISO 10272-1/2017 [41]. Procedure B was used to detect *Campylobacter* by enrichment, in samples with low numbers of *Campylobacter* and high level of background microflora. Briefly, samples were added to the liquid enrichment medium (Preston broth) (Condalab, Madrid, Spain) and incubated in a microaerobic atmosphere at 41.5 °C for 24 h. Then, the enrichment culture obtained was inoculated into selective Modified Charcoal Cefoperazone Deoxycholate (mCCD) agar (Oxoid, Hampshire, UK). The suspect *Campylobacter* colonies were identified by MALDI-TOF.

MRSA was isolated according to EURL-AR protocol [42] that consists of a pre-enrichment with Mueller-Hinton broth containing 6.5% sodium chloride (NaCl) (Oxoid, Hampshire, UK) and incubated at 35–37 °C for 16–24 h. Thereafter, a 10 mL loopful of the broth was spread on Brilliance MRSA 2 agar (Oxoid, Hampshire, UK) and incubate at 35–37 °C for 16–24 h. Suspected colonies were confirmed by MALDI-TOF and then purified. Multiplex PCR was used to amplify methicillin resistance *mecA*, *mecC* genes, *Staphylococcal complement inhibitor gene* (*scn*), Panton–Valentine Leukocidin (PVL), and Livestock-associated MRSA clonal complex (CC397) gene, according to EURL-AR protocol. As amplification of the resistance genes was not performed, the designation-presumptive MRSA was used.

### 2.6. Ethics

Biological samples were collected with the permission of farmers and according to good veterinary practices and animal welfare standards. Experimental procedures were performed according to the European Directive 2010/63/EU on the protection of animals used for scientific purposes and were approved by the Órgão para o Bem-Estar Animal (ORBEA) of Escola Superior Agrária de Viseu (ESAV) with the reference 01/ORBEA/2024. The ethics committee of Instituto Politécnico de Viseu (IPV) approved the data collection instrument (questionnaire survey), and protocol (50/SUB/2023).

### 2.7. Statistical Analysis

Data collected through questionnaires and microbiological data were entered into a database (Microsoft Excel 2016^®^, Microsoft Corp., Redmond, WA, USA). Statistical analyses were performed using IBM SPSS Statistics, version 28.0.0.0 (IBM Corp., Armonk, NY, USA, 2020) and included both descriptive and inferential methods. The chi-square test of independence was initially used to explore potential relationships between categorical variables. A level of significance of 5% was applied throughout the analysis. Cluster analysis was conducted in two stages: first, the two-step cluster method was applied to explore the natural grouping structure in the data and to determine the optimal number of clusters; subsequently, the k-means clustering algorithm was used to refine the classification of farms into groups with similar characteristics. These clusters were then used as the dependent variable in a multinomial logistic regression model, developed to explain the distribution of fecal and nasal carriage of potentially zoonotic bacteria among small ruminant farms in Portugal’s Central Region. In the regression model, only statistically significant variables (*p* < 0.05) were retained.

## 3. Results

### 3.1. Farm Demographic Characterization

A total of 122 farms from Portugal’s Central Region participated in the study. Of these farms, 72 (59.0%) were sheep farms and 50 (41.0%) were goat farms. The number of animals ± standard deviation of the sampled sheep farms was 158.2 ± 366.4 (minimum = 7, maximum = 3000), and goat farms was 99.9 ± 141.2 (minimum = 6, maximum = 800).

Most of the farmers were male (74.6%), had completed nine years of schooling or less (64.8%), were over 40 years old (68.0%), and had over 20 years of professional experience (45.9%). The scores of biosafety practices, and of farmers’ knowledge, attitudes and practices regarding the use of antibiotics obtained through the KAP questionnaire were 7.6 ± 2.7 (mean ± standard deviation) (scale 1–17), and 4.61 ± 1.8, 6.0 ± 1.1, and 4.4 ± 1.8 (scale 0–7), respectively (Supplementary Table S2—Biosafety and KAP responses). Most farmers reported that they administered antibiotics to their animals (96.7%) and stored antibiotics on their farm (87.7%), mainly oxytetracycline (81.1%), penicillin plus di-hidrostreptomycin (6.6%), and ampicillin/amoxicillin (4.1%). These antibiotics are primarily used to treat mastitis (39.3%), foot root (39.3%), and diarrhea (36.1%).

The sampled farms were mainly located in the districts of Guarda (36.9%) and Viseu (26.2%), and their primary purpose was meat production (50.8%). Most farms practiced conventional animal production (89.3%), and the animals were raised mainly in a semi-extensive system (77.9%) (Table 1).

Most of the sampled animals were female (716; 97.8%), aged between two and six years (382; 52.2%). Most had no defined breed (69.0%), although, the sample included sheep and goats from imported and autochthonous breeds (Table 2).

### 3.2. Individual-Level Prevalence of Zoonotic Enteric Bacteria

A total of 732 fecal samples were screened for the presence of *Campylobacter* spp., *Salmonella* spp. and presumptive ESBL-producing strains of *E. coli.* Of these samples, 432 (59.0%) were from sheep and 300 (41.0%) from goats.

Commensal *E. coli* was isolated from all the fecal samples. The overall prevalence of *Campylobacter* spp. was 15.6%. The prevalence was significantly higher in sheep (19.0%) than in goats (10.7%) (*p* = 0.001). Three *Campylobacter* species were identified by MALDI-TOF. Overall, *C. jejuni* (10.4%) was more frequently isolated than *C. coli* (4.0%). *C. fetus* was isolated only in one fecal sample. Both *C. jejuni* and *C. coli* were isolated more frequently from sheep feces (13.8% and 4.9%, respectively) than goat feces (5.3% and 2.6%, respectively). The overall prevalence of *Salmonella* spp. was 8.3%, significantly higher in sheep (12.7%) than in goats (2.0%) (*p* < 0.001). The overall prevalence of presumptive ESBL-producing strains of *E. coli* was 5.2% (Table 3).

### 3.3. Farm-Level Prevalence of Presumptive Methicillin-Resistant S. aureus

A total of 122 pools of nasal swabs, consisting of 72 sheep pools and 50 goat pools were analyzed. Of the farms analyzed, seven farms (5.7%) were presumptively MRSA positive, including four goat farms (8.0%) and three sheep farms (4.2%). The gene-encoding protein A (*spa*) was PCR amplified in one sheep farm and three goat farms. The genes *mecA* methicillin resistance was PCR amplified in one sheep farm and the gene *mecC methicilin resistance* was PCR amplified in one sheep and one goat farms. The *Staphylococcal complement inhibitor* gene (*scn*), the Panton–Valentine Leukocidin (PVL) gene and the livestock-associated MRSA clonal complex (CC398) gene were not amplified in any presumptive MRSA isolates (Table 4).

### 3.4. Characterization of Positive Farms

The prevalence of *Campylobacter* spp., *Salmonella* spp., and presumptive ESBL-producing strains of *E. coli* and presumptive MRSA strains were determined at the farm level.

The overall prevalence of fecal carriage of *Campylobacter* spp. at farm level was 45.1% (55/122). Sheep farms (52.8%; 38/72) were more frequently considered positive than goat farms (34.0%; 17/50, *p* = 0.031). Fecal carriage of *Campylobacter* spp. was significantly different among production systems (*p* < 0.001) since nearly all *Campylobacter* spp. positive farms practiced a semi-extensive system of production (92.7%).

*Salmonella* spp. was isolated from 35 out of 122 farms (28.7%), 30 of which were sheep farms and five of which were goat farms. Sheep farms (41.7%; 30/72) were more frequently positive than goat farms (10.0%; 5/50, *p* < 0.001).

The overall prevalence of presumptive ESBL-producing strains of *E. coli* at the farm level was 15.6% (19/122). The proportion of positive sheep and goat farms was 16.7% (12/72) and 14.0% (7/50), respectively. The prevalence of presumptive ESBL-producing strains of *E. coli* was statistically different among districts (*p* < 0.001) and production systems (*p* = 0.014). The highest proportion of positive farms was in the Leiria district (36.8%) and practiced a semi-extensive production system (52.6%).

The overall prevalence of presumptive MRSA was 5.7% (7/122), with 4.2% (3/72) in sheep farms and 8.0% (4/50) in goat farms. The prevalence of presumptive MRSA positive farms was statistically different among districts (*p* < 0.001), and production systems (*p* < 0.001). Prevalence was higher in farms located in the districts of Coimbra and Leiria (42.9%), and with semi-extensive production system (42.9%) (Table 5).

### 3.5. Cluster Analysis

A cluster analysis was performed to identify farm clusters with similar characteristics based on the isolation of *Campylobacter* spp., *Salmonella* spp., presumptive ESBL-producing strains of *E. coli*, and presumptive MRSA. All predictors included in the model showed high relative importance values (>0.8), indicating strong contributions to the differentiation of clusters. The solution suggested by the two-step procedure consisted of three clusters, with a silhouette coefficient of 0.9, reflecting excellent cohesion and separation of the groups. To assess the stability and robustness of this classification, a k-means clustering algorithm was subsequently applied. The same three-cluster structure was reproduced, thereby confirming the consistency of the results.

Cluster 1 (“Resistant”) comprised 49 (40.2%) of the farms in which presumptive ESBL-producing strains of *E. coli* was isolated in 18.4% and presumptive MRSA was isolated in 12.2% of the farms.

Cluster 2 (“Campylobacter”) consisted of 38 (31.1%) of the farms in which *Campylobacter* spp. was isolated in all cases (100%), together with presumptive MRSA (2.6%) and presumptive ESBL-producing strains of *E. coli* (7.9%).

Cluster 3 (“Salmonella”) comprised 35 (28.7%) of the farms in which *Salmonella* spp. was isolated in all cases (100%), together with *Campylobacter* spp. (48.6%) and presumptive ESBL-producing strains of *E. coli* (20.0%).

**Figure 1:**
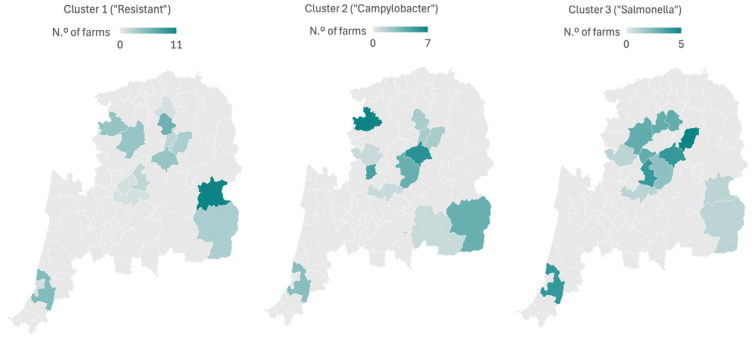
Geographical distribution of groups of farms (clusters) with similar characteristics based on the isolation of *Campylobacter* spp., *Salmonella* spp., presumptive ESBL-producing strains of *E. coli*, and presumptive MRSA. The number of farms is shown by color gradient, as indicated in the legend.

The proportion of positive farms for *Salmonella* spp. (*p* < 0.001), *Campylobacter* spp. (*p* < 0.001), and presumptive MRSA (*p* = 0.018) were statistically different among the three clusters (Figure 1, Table 6).

### 3.6. Multinomial Logistic Regression Model

A multinomial logistic regression was performed to investigate the association between farmers’ characteristics and cluster membership. The dependent variable was the three-cluster solution obtained from the k-means analysis, with the “Resistant” cluster set as the reference category. Independent variables included farmers’ attitudes, practices, knowledge, and biosafety (treated as continuous predictors). The categorical covariates were: farmer age (≤40 vs. >40 years, reference ≥ 40), farming experience (≤20 vs. >20 years, reference ≥ 20), animal species (sheep vs. goats, reference = sheep), district of the farm (Castelo Branco, Coimbra, Guarda, Leiria, and Viseu, with Viseu as the reference), and the presence of stored ampicillin/amoxicillin (absence vs. presence, with presence as the reference).

The model estimated the relative risk ratios of belonging to each cluster compared with the reference cluster, while controlling for all covariates. Statistical significance was set at *p* < 0.05. Farmers’ practices, district, and animal species emerged as significant predictors of cluster allocation, whereas age, farming experience, farmers’ knowledge, biosafety, and the presence of stored antimicrobials were not statistically significant.

#### 3.6.1. Odds of “Campylobacter” Cluster Compared to “Resistant” Cluster

The probability of a farm belonging to the “Campylobacter” cluster, compared to the “Resistant” cluster, was significantly associated with animal species, farm district and farmer’s practices regarding the antibiotic use. Goat farms were 66.9% less likely to be classified in the “Campylobacter” cluster (compared to the “Resistant” cluster) compared to sheep farms. Farms located in Castelo Branco district were 87.7% less likely to be classified in the “Campylobacter” cluster (compared to the “Resistant” cluster) compared to farms located in Viseu district. Higher scores in farmers’ practices regarding antibiotic use were associated with a reduced likelihood of belonging to the “Campylobacter” cluster compared to the “Resistant” cluster. For each one-point increase in the score, the odds of being in the “Campylobacter” cluster decreased by 29.6%.

Farms located in Coimbra, Guarda and Leiria districts were 81.9%, 16.5% and 71.7% less likely to be classified in the “Campylobacter” cluster (compared to the “Resistant” cluster), respectively, compared to farms located in Viseu district, although the association is not statistically significant.

#### 3.6.2. Odds of “Salmonella” Cluster Compared to “Resistant” Cluster

The probability of a farm belonging to the “Salmonella” cluster, in comparison to the “Resistant” cluster, was significantly associated with animal species, farm district and farmer’s practices regarding antibiotic use.

Goat farms were 92.5% less likely to belong to the “Salmonella” cluster (compared to “Resistant” cluster) compared to sheep farms. Farms located in Castelo Branco district were 94.0% less likely to be classified in the “Salmonella” cluster (compared to the “Resistant” cluster) compared to farms located in Viseu district. Farmers’ practices regarding antibiotic use were associated with a reduced likelihood of farms belonging to the “Salmonella” cluster compared to the “Resistant” cluster. For each one-point increase in the score, the odds of being in the “Salmonella” cluster decreased by 26.3%.

Farms located in Coimbra, Guarda and Leiria districts were 10.8%, 16.7% and 61.1% less likely to be classified in the “Salmonella” cluster (compared to the “Resistant” cluster), respectively, compared to farms located in Viseu district, although the association is not statistically significant (Table 7).

## 4. Discussion

This study reports the prevalence of *Campylobacter* spp., *Salmonella* spp., and presumptive ESBL-producing strains of *E. coli* in the feces of small ruminants from Portugal’s Central Region for the first time. The study surveyed an adequate sample of small ruminants (432 sheep and 300 goats) from this region.

Portugal’s Central Region contributes with 23% of sheep milk and 35% of goat milk produced in Portugal contributes with about one-third of the national production of small ruminants used for meat and milk production. Small ruminants are predominantly reared in a semi-extensive production system, family-based, sometimes on household premises. The small-scale dairy farms still practice hand milking, promoting close contact between animals and farm workers. Furthermore, this region is recognized by the production of raw milk cheeses (artisanal and industrial methods). These cheeses are highly appreciated because of their unique taste and texture. However, raw milk cheese consumption carries potential public health concerns due to the presence of *S. aureus*, *E. coli*, *Listeria monocytogenes*, and *Salmonella* spp. [43].

Research on the prevalence of *Campylobacter* spp., *Salmonella* spp., and *E. coli* in small ruminants are limited, but it is essential for understanding the role of sheep and goats as reservoirs of these zoonotic bacteria. The prevalence of the fecal carriage of these Enterobacteriaceae among small ruminants indicates their potential to contaminate animal products, other animals, humans, and the environment [44,45].

In this study, the prevalence of fecal carriage of *Campylobacter* spp. (15.6%) was significantly higher in sheep (19.0%) than in goat feces (10.7%). Several studies reported *Campylobacter* spp. prevalences ranging from 5% to 80.7% in small ruminants [44,46,47,48,49,50,51]. Differences observed across studies and countries could be attributed to several factors related to animal species, animal health, environment and farm management. Similar to other worldwide studies, our research suggests that small ruminants, particularly sheep, from Portugal’s Central Region play an important role as reservoir of *Campylobacter* spp. and potential risk for human infections. The transmission of foodborne pathogens (or enteric pathogens) from the animal reservoir to humans occurs predominantly through the consumption of food and water contaminated with feces. Contamination of meat products typically occurs within the slaughterhouse, with the cross-contamination with *Campylobacter* spp. of the carcass occurring during the slaughter or cutting. This contamination can occur either directly or indirectly through the hands and equipment of slaughterhouse workers. In addition, humans may be infected by direct contact with animals or contaminated environments with animal feces. In the present study, *C. jejuni*—the most important cause of human bacterial gastroenteritis in the industrialized world—was more frequently isolated than *C. coli*, as reported in other studies [44,48,51,52,53]. The fecal carriage of *Campylobacter* spp. was significantly higher in semi-extensive than in intensive/semi-intensive or extensive management systems, which contradicts a previous study that reported high frequency of isolation from semi-intensively managed animals in Trinidade [46]. The findings of our study may be explained by the imbalanced sample, which is mainly composed of farms with a semi-extensive management system (77.9%).

Regarding *Salmonella* spp., the overall prevalence of fecal carriage was 8.3%, in this study, which was significantly higher in sheep (12.7%) than in goats (2.0%). A meta-analysis study reported lower prevalence in sheep and goat guts, in Africa, 4.5% and 2.2%, respectively [50]. In contrast, the prevalence of *Salmonella* spp. from fecal samples obtained from slaughterhouses in the United States, Bahamas and Mexico was higher (13,91%), ranging from 10.3% (goats), 11.4% (lambs) and 42.0% (mixed pens) [43]. A recent meta-analysis based on 49 studies reported a global prevalence of 8.3% and 7.0% in sheep and goats, respectively, in samples of carcass, fecal content and lymph nodes collected at the slaughterhouses [54]. Asymptomatic carriers of *Salmonella* spp., namely poultry, pigs, and cattle can be a source of contamination to humans. Reports of the European Food Safety Authority (EFSA) and European Centre of Disease Prevention and Control (ECDC) identified salmonellosis as the main cause of foodborne outbreaks [11,12].

In the European Union, the monitoring of AMR in *E. coli* from livestock and retail meat samples is mandatory [55]. However, small ruminants are not included in the official Portuguese AMR surveillance program. The significance of small ruminant production for rural communities, in terms of both socioeconomics and the proximity between humans and animals, reinforces the need for an AMR surveillance program for these species. The overall prevalence of the fecal carriage of presumptive ESBL-producing strains of *E. coli* was 5.2%, with no statistically significant differences observed between sheep and goats. The first report of fecal carriage of ESBL-producing strains of *E. coli* in healthy sheep (5% prevalence) in Portugal was carried out at slaughterhouse from the Central region of the country in 2013 [56]. A recent study carried out in 2021, reported a prevalence of fecal carriage of ESBL-producing strains of *E. coli* of 90.5% on a sheep farm in the southern of Portugal where animals were reared in an extensive grazing management system. The majority of isolates showed resistance to non-beta-lactam antibiotics, specifically tetracycline and sulfamethoxazole-trimethoprim [57]. These results were unexpected since less intensive management systems have been associated with lower prevalence of cefotaxime-resistant *E. coli* [58]. The historical farm records of antibiotic administration could help to understand this high prevalence of ESBL in sheep; however, such information was not available [57]. In other countries, including Chile, Pakistan, Switzerland, Tanzania, Turkey, the United Kingdom, Spain, and South Africa the prevalences of ESBL-producing strains of *E. coli* in sheep ranged from 1.5% to 28.8% [25,59,60,61,62,63,64].

A study carried out in Northern Spain obtained a prevalence of presumptive ESBL/AmpC-producing *E. coli* of 7.0% at farm level in sheep. While the animal level prevalence in our study agreed with previous studies, the farm level prevalence was higher, with 14.0% in goats and 16.7% in sheep farms. Although low prevalence rates of ESBL-producing *E. coli* strains are expected, given that ARG have been present in nature long before antibiotic use, their massive application, together with the ability of *E. coli* to accumulate ARG by horizontal transfer, has increased the prevalence of ESBL-producing *E. coli* strains [19]. Differences in the prevalence rates of ESBL-producing *E. coli* between studies can be attributed to disparities in sampling strategies and isolation methods, which makes comparisons difficult. Factors related to the management system (intensive *versus* extensive), and AMU can help explain the differences among studies.

In the present study, presumptive MRSA was isolated from nasal samples collected from six animals from each farm. The overall prevalence was 5.7%, higher in goat farms (8.0%) than in sheep farms (4.2%). Care should be taken when comparing this study with others, given the differences in experimental design, namely sampling (individual vs. pool samples), *S. aureus* isolation and MRSA identification (phenotypic culture-based vs. PCR), which inevitably influenced the results. For instance, the isolation rate from individual nasal swabs of small ruminants in Nigeria was 7.5% [65], in India was 9.5% [66], and in Egypt was 3.8% in sheep and 3.9% in goats [67]. In addition to experimental design, other factors such as climatic conditions, management system (intensive vs. extensive) of the farm and the extent of prophylactic and therapeutic use of antibiotics may influence nasal carriage of MRSA [30,65,68]. The nasal carriage of *S. aureus* and MRSA represents an indicator of colonization and a potential risk of infection for people who had direct or indirect contact with them and with their shared environments [30,69].

Three sheep and four goat *S. aureus* isolates grew on Brillance medium, demonstrating AMR. However, the *mecA* gene was PCR amplified in only one sheep MRSA isolate and *mecC* gene was amplified in one sheep and one goat presumptive MRSA isolate, indicating the presence of resistance mechanisms other than *mecA* and *mecC* genes. Although *mecA* and *mecC* are the most frequently identified PBP2a-encoding genes responsible for *S. aureus* AMR in livestock animals, two less frequent *mec* genes, *mecB* and *mecD*, have already been described [19]. In addition, other resistance mechanisms, such as efflux pumps, could justify the grow of *S. aureus* isolates on MRSA-selective medium.

CC398 is the most common LA-MRSA strain worldwide and in Europe, but the MRSA epidemiology has changed significantly in the last years, as several clonal complexes and subtypes within the clonal complexes have been identified in small ruminants [19,70], which may justify the absence of detection in our isolates. In addition, to the CC398, which was observed in a single isolate, other CCs have been identified in small ruminants [19]. Genes of the human specific immune evasion cluster (IEC) are considered a marker of *S. aureus* adaptation to human host. Specifically, the *scn* gene that encodes a staphylococcal complement inhibitor is considered a functionally essential marker of IEC-positive isolates [71]. PVL is a cytolysin that can produce tissue necrosis and leukocyte destruction and is frequently detected in *S. aureus* isolates from patients with deep skin and soft tissue infections. None of these genes, *scn* and PVL were detected in our isolates, indicating absence of human adaptation.

A cluster analysis was conducted, followed by a multinomial logistic regression to assess the distribution of fecal and nasal carriage of presumptive ESBL-producing strains of *E. coli* and presumptive MRSA among small ruminant farms in Portugal’s Central Region. The findings of this study indicate that goat farms were less likely to belong to the “Campylobacter” and “Salmonella” clusters (compared to “Resistant” cluster) compared to sheep farms. In other words, compared to sheep farms, goat farms were more likely to belong to the “Resistant” cluster than to the “Campylobacter” and “Salmonella” clusters, which included antimicrobial resistant *E. coli* and *S. aureus*. Several studies have already documented higher prevalences of *Campylobacter* spp. [8,51] and *Salmonella* spp. [43,50,54] isolation in sheep than goats, probably reflecting species predisposition, related to feeding behavior. Sheep are mainly grazers, feeding primarily on low vegetation that grows close to the ground, while goats are more likely to browse, feeding on shrubs, branches, leaves, and taller plants, which reduces the likelihood of fecal–oral transmission of these Enterobacteriaceae. Given that the primary factor contributing to the emergence of AMR is the misuse of antibiotics, it is logical to hypothesize that AMU is more prevalence in goat farms than in sheep farms in Portugal’s Central Region. Interestingly, a higher proportion of sampled goat farms (36.0%) than sheep farms (15.3%) were raised for milk production.

Antibiotics are frequently used on dairy farms to control mastitis [72,73] caused by *S. aureus* and coagulase-negative *Staphylococcus* [74,75]. Furthermore, several studies suggest the potential horizontal transfer of antibiotic resistance genes from coagulase-negative *Staphylococcus* to *S. aureus* [76,77]. Therefore, we can hypothesize that the higher prevalence of MRSA in goat farms than in sheep farms may be attributed to AMU to control mastitis, associated with the intensification of livestock farming [78] that is observed in goat dairy production in this region. *E. coli* is a frequent cause of intramammary infection in small ruminants [79,80], as well as an important cause of diarrhea in young animals [81]. Diarrheal diseases account for a significant AMU in small ruminant farms. However, given that genes that encode ESBL enzymes can be found on plasmids or on single chromosomes of bacteria, resistance determinants can be horizontally transferred between bacteria from different species [22].

The risk of belonging to “Campylobacter” and “Salmonella” clusters (compared to “Resistant” cluster) was associated to farm location. Compared to farms located in Viseu district, farms from Castelo Branco district were more likely to belong to “Resistant” cluster, which may be related to production system, namely farming intensification, access to medicines outside the official marketing circuit, among others. It is worth noting that most of the farmers who participated in this study administered antibiotics to their animals (96.7%), had antibiotics stored on the farm and did not record antibiotic use in the medication book (62.3%), reinforcing the easy access to antibiotics and the lack of traceability of their use in Portugal’s Central Region.

Regarding farm locations, it should be noted the relative proximity of farms in each cluster, which suggests animal movement and/or fomite transfer. Indeed, in this region, physical biosafety measures designed to prevent the entry of pathogenic microorganisms into the farm were not widely implemented. For example, farm access remained open on 45.1% of the farms, there was no functional rotary wheel wash for vehicle disinfection on 97.5% of the cases, and employees and visitors did not change their shoes and clothing upon entering the farm on 66.4% and 97.5% of the cases.

Knowledge, attitudes and beliefs about antimicrobials drives their use [82]. In this study, farmers’ practices regarding antibiotic use were associated with a reduced likelihood of farms belonging to the “Campylobacter” and “Salmonella” clusters compared to the “Resistant” cluster. That is, farms belonging to farmers that stated best practices regarding antibiotic use were more likely to belong to “Resistant” cluster. These apparently contradictory findings could be explained by information (or questionnaire) bias as the in-person survey with a self-report of knowledge, attitude and practices can inadvertently cause participant to offer answers that they anticipate the researcher’s views as correct or favorable, which may affect the accuracy and integrity of the data [83].

In addition to the drawbacks of questionnaire data collection already mentioned, other limitations of this study should be considered, namely the sampling methodology, which included convenience selection of farms and the inclusion of a fixed number of animals per farm, regardless of livestock density. The field conditions encountered did not allow sample size calculation for each farm. Furthermore, inferential statistical analysis was performed considering both sheep and goat farms to increase sample size and consequently statistical robustness, therefore, it was not possible to identify risk factors related to zoonotic bacteria carriage specifically associated to animal species (sheep *versus* goats).

However, this study reinforces the role of small ruminants as asymptomatic reservoirs of *Campylobacter* spp., *Salmonella* spp., *E. coli* and *S. aureus* and confirms, for the first time, their role as carriers of presumptive antibiotic-resistant zoonotic bacteria in the Central Region of Portugal. Nevertheless, to better understand the impact of small ruminants as a reservoir of antibiotic-resistant zoonotic bacteria, future studies should focus on the susceptibility/resistance profile of the isolates obtained, as well as on the GRA involved.

## 5. Conclusions

The overall prevalence of *Campylobacter* spp. and *Salmonella* spp. was 15.6% and 8.3%, respectively, being significantly higher in sheep (19.0% and 12.7%) than goats (10.7% and 2.0%). Presumptive ESBL-producing strains of *E. coli* was isolated from 5.2% of the animals, without statistically significant differences between sheep (5.3%) and goats (5.0%). Presumptive MRSA was isolated from 5.7% of the farms, with a prevalence of 4.2% in sheep and 8.0% in goat farms (8.3%), but the *mecA* and *mecC* genes PCR amplified in only 0.8% and 1.6% of the presumptive MRSA isolates obtained.

The likelihood of farms belonging to “Campylobacter” and “Salmonella” clusters in comparison to “non-resistant” cluster was significantly associated with animal species, farm location and farmers’ practices regarding antibiotic use.

## Figures and Tables

**Table 1:** Characterization of sampled sheep and goat farms collected in a cross-sectional study in Portugal’s Central Region between 29 April 2024 and 13 March 2025.

Characteristics	Category	Sheep (n = 72) n (%)	95% CI	Goats (n = 50) n (%)	95% CI	Total (n = 122) n (%)	95% CI
District	Castelo Branco	14 (19.4)	0.1020–0.2859	7 (14.0)	0.0438–0.2362	21 (17.2)	0.1052–0.2391
	Coimbra	7 (9.7)	0.0288–0.1656	4 (8.0)	0.0048–0.1552	11 (9.0)	0.0393–0.140
	Guarda	26 (36.1)	0.2502–0.4721	19 (38.0)	0.2455–0.5145	45 (36.9)	0.2832–0.4545
	Leiria	8 (11.1)	0.03852–0.1837	5 (10.0)	0.0168–0.1831	13 (10.7)	0.0518–0.1613
	Viseu	17 (23.6)	0.1380–0.3342	15 (30.0)	0.1730–0.4270	32 (26.2)	0.1842–0.3403
Production purpose	Milk	11 (15.3)	0.0697–0.2359	18 (36.0)	0.2270–0.4930	29 (23.8)	0.1622–0.3132
	Meat	44 (61.1)	0.4985–0.7237	18 (36.0)	0.2270–0.4930	62 (50.8)	0.4195–0.5969
	Milk + Meat	17 (23.6)	0.1380–0.3342	12 (24.0)	0.1216–0.3584	29 (23.8)	0.1622–0.3132
	Other *	2 (2.8%)	0.000–0.0657	2 (4.0)	0.000–0.0943	2 (1.6)	0.0000–0.0389
Production type	Conventional	65 (90.3)	0.8343–0.9712	44 (88.0)	0.7899–0.9701	109 (89.3)	0.8387–0.9482
	Biological/organic	7 (9.7)	0.0288–0.1656	6 (12.0)	0.0299–0.2101	13 (10.7)	0.0518–0.1613
Production system	Intensive + Semi-intensive	0		8 (16.0)	0.0584–0.2616	8 (6.6)	0.0216–0.1095
	Extensive	12 (16.7)	0.0806–0.2527	7 (14.0)	0.0438–0.2362	19 (15.6)	0.0914–0.2201
	Semi-extensive	60 (83.3)	0.7472–0.9194	35 (70.0)	0.5730–0.8270	95 (77.9)	0.7050–0.8523

* Other—Land clearing, wool, among others; CI—Confidence Interval.

**Table 2:** Characterization of sampled sheep and goats (n = 732), collected in a cross-sectional study in Portugal’s Central Region between 29 April 2024 and 13 March 2025.

Characteristics	Category	Sheep (n = 432) n (%)	95% CI	Goat (n = 300) n (%)	95% CI	Total (n = 732) n (%)	95% CI
Sex	Female	424 (98.1)	0.96–0.99	292 (97.3)	0.96–0.99	716 (97.8)	0.97–0.99
	Male	8 (1.9)	0.005–0.03	8 (2.7)	0.008–0.04	16 (2.2)	0.01–0.03
Age (months)	0–9	12 (2.8)	0.012–0.043	18 (6.0)	0.033–0.0879	30 (4.1)	0.0266–0.0553
	10–24	76 (17.6)	0.14–0.21	75 (25.0)	0.201–0.299	151 (20.6)	0.1770–0.2356
	25–72	382 (51.4)	0.85–0.91	160 (53.3)	0.477–0.590	382 (52.2)	0.4857–0.5580
	73–120	110 (25.5)	0.21–0.29	42 (14.0)	0.1007–0.1793	152 (20.8)	0.1783–0.2370
	>120	12 (2.8)	0.012–0.043	5 (1.7)	0.0022–0.0311	17 (2.3)	0.0123–0.0341
Breed	No defined breed	269 (62.3)	0.577–0.668	236 (78.7)	0.7403–0.8330	505 (69.0)	0.6563–0.7234
	Serra da Estrela	135 (31.3)	0.269–0.356	-		135 (18.4)	0.2563–0.2125
	Suffolk	6 (1.4)	0.0028–0.0249	-		6 (0.8)	0.0017–0.0147
	Charolês x Suffolk	6 (1.4)	0.0028–0.0249	-		6 (0.8)	0.0017–0.0147
	Merino Preto	12 (2.8)	0.012–0.043	-		12 (1.6)	0.0072–0.0256
	Churra do Campo	4 (0.9)	0.0002–0.0183	-		4 (0.5)	0.0001–0.0108
	Saanen	-		8 (2.7)	0.008–0.04	8 (1.3)	0.0034–0.0185
	Serpentina	-		6 (2.0)	0.0042–0.0358	6 (0.8)	0.0017–0.0147
	Murciana	-		16 (5.3)	0.0279–0.0788	16 (2.2)	0.0113–0.0324
	Crossed Serrana	-		16 (5.3)	0.0279–0.0788	16 (2.2	0.0113–0.0324
	Jarmelista	-		6 (2.0)	0.0042–0.0358	6 (0.8)	0.0017–0.0147
	Alpina	-		6 (2.0)	0.0042–0.0358	6 (0.8)	0.0017–0.0147
	Charnequeira	-		6 (2.0)	0.0042–0.0358	6 (0.8)	0.0017–0.0147

CI—Confidence Interval.

**Table 3:** Prevalence of fecal carriage of zoonotic enteric bacteria in sheep and goats collected in a cross-sectional study in Portugal’s Central Region between 29 April 2024 and 13 March 2025.

Bacteria	Sheep (n = 432) n (%)	95% CI	Goats (n = 300) n (%)	95% CI	*p* ^2^	Total (n = 732) n (%)	95% CI
*Campylobacter* spp.	82 (19.0)	0.1528–0.2268	32 (10.7)	0.0717–0.1416	0.001	114 (15.6)	0.1295–0.1821
*C. jejuni*	60 (13.8)	0.1063–0.1715	16 (5.3)	0.0279–0.0788	-	76 (10.4)	0.0817–0.1259
*C. coli*	21 (4.9)	0.0283–0.0689	8 (2.6)	0.0084–0.0449	-	29 (4.0)	0.0255–0.0537
*C. fetus*	1 (0.3)	0.0000–0.0068	-		-	1 (0.1)	0.0000–0.0040
*Salmonella* spp.	55 (12.7)	0.0959–0.1587	6 (2.0)	0.0042–0.0358	<0.001	61 (8.3)	0.0633–0.1034
ESBL *E. coli* ^1^	23 (5.3)	0.0321–0.0744	15 (5.0)	0.0253–0.0745	-	38 (5.2)	0.0358–0.0680

^1^ Extended spectrum β-lactamases (ESBLs)-producing strains of *Escherichia coli* isolated in MacConkey Agar supplemented with cefotaxime; ^2^ Fisher exact test; CI—Confidence Interval.

**Table 4:** Prevalence and genetic characterization of presumptive MRSA isolated from pooled nasal swabs of sheep and goats collected in a cross-sectional study in Portugal’s Central Region, between 29 April 2024 and 13 March 2025.

Bacteria	Sheep (n = 72) n (%)	95% CI	Goats (n = 50) n (%)	95% CI	Total (n = 122) n (%)	95% CI
Presumptive MRSA ^1^	3 (4.2)	0.0000–0.0878	4 (8.0)	0.0048–0.1552	7 (5.7)	0.0161–0.0986
*mecA* ^2^	1 (1.4)	0.0000–0.0409	0		1 (0.8)	0.0000–0.0242
*mecC* ^2^	1 (1.4)	0.0000–0.0409	1 (2.0)	0.0000–0.0588	2 (1.6)	0.0000–0.389
spa ^2^	1 (1.4)	0.0000–0.0409	3 (6.0)	0.0000–0.1258	4 (3.3)	0.0012–0.0644
scn ^2^	0		0		0	
CC398 ^2^	0		0		0	
PVL ^2^	0		0		0	

^1^ Methicillin-resistant *Staphylococcus aureus* isolated on Brilliance MRSA 2 agar that contains an antibiotic cocktail; ^2^ Multiplex PCR; CI—Confidence Interval.

**Table 5:** Characterization of positive farms for zoonotic bacteria in Portugal’s Central Region.

Characteristics		*Campylobacter* spp.		*Salmonella* spp.		Presumptive *E. coli* ESBL		Presumptive MRSA	
		Negative	Positive	Negative	Positive	Negative	Positive	Negative	Positive
		(n = 67) n (%)	(n = 55) n (%)	(n = 87) n (%)	(n = 35) n (%)	(n = 103) n (%)	(n = 19) n (%)	(n = 115) n (%)	(n = 7) n (%)
Species	*p*	0.031		<0.001		>0.05		>0.05	
	Sheep	34 (50.7)	38 (69.1)	42 (48.3)	30 (85.7)	60 (58.3)	12 (63.2)	69 (60.0)	3 (42.9)
	Goat	33 (49.3)	17 (30.9)	45 (51.7)	5 (14.3)	43 (41.7)	7 (36.8)	46 (40.0)	4 (57.1)
Species association	*p*		>0.05		0.015	>0.05		>0.05	
	Only sheep	27 (40.3)	31 (56.3)	36 (41.4)	22 (62.9)	48 (46.6)	10 (52.6)	55 (47.8)	3 (42.9)
	Only goat	23 (34.3)	12 (21.8)	32 (36.8)	3 (8.6)	29 (28.2)	6 (31.6)	31 (27.0)	4 (57.1)
	Sheep + goat	17 (25.4)	11 (20.0)	18 (20.7)	10 (28.6)	25 (24.3)	4 (21.1)	28 (24.3)	0
	Other	0	1 (1.8)	1 (1.1)	0	1 (1.0)	0	1 (0.9)	0
District	*p*	>0.05		>0.05		<0.001		<0.001	
	Castelo Branco	15 (22.4)	6 (10.9)	19 (21.8)	2 (5.7)	16 (15.5)	5 (26.3)	20 (17.4)	1 (14.3)
	Coimbra	6 (9.0)	5 (9.1)	6 (6.9)	5 (14.3)	11 (10.7)	0	8 (7.0)	3 (42.9)
	Guarda	22 (32.8)	23 (41.8)	31 (35.6)	14 (40.0)	41 (39.8)	4 (21.1)	45 (39.7)	0
	Leiria	10 (14.9)	3 (5.5)	9 (10.3)	4 (11.4)	6 (5.8)	7 (36.8)	10 (8.7)	3 (42.9)
	Viseu	14 (20.9)	18 (32.7)	22 (25.3)	10 (28.6)	29 (28.2)	3 (15.8)	32 (27.8)	0
Production purpose	*p*	>0.05		>0.05		>0.05		>0.05	
	Milk	20 (29.9)	9 (16.4)	20 (23.0)	9 (25.7)	27 (26.2)	2 (10.5)	26 (22.6)	3 (42.9)
	Meat	31 (46.3)	31 (56.4)	47 (54.0)	15 (42.9)	50 (48.5)	12 (63.2)	59 (51.3)	3 (42.9)
	Milk + Meat	14 (20.9)	15 (27.3)	18 (20.7)	11 (31.4)	24 (23.3)	5 (26.3)	28 (24.3)	1 (14.3)
	Other *		0	2 (2.3)	0	2 (1.9)	0	2 (1.7)	0
Production type	*p*	>0.05		>0.05		>0.05		>0.05	
	Conventional	57 (85.1)	52 (94.5)	76 (87.4)	33 (94.3)	92 (89.3)	17 (89.5)	103 (89.6)	6 (85.7)
	Biological/organic	10 (14.9)	3 (5.4)	11 (12.6)	2 (5.7)	11 (10.7)	2 (10.5)	12 (10.4)	1 (14.3)
Production system	*p*	<0.001		>0.05		0.014		<0.001	
	Intensive/Semi-intensive	8 (11.9)	0	8 (9.2)	0	5 (4.8)	3 (15.8)	6 (5.2)	2 (28.6)
	Extensive	15 (22.4)	4 (7.3)	16 (18.4)	3 (8.6)	13 (12.6)	6 (31.6)	17 (14.8)	2 (28.6)
	Semi-extensive	44 (65.7)	51(92.7)	63 (72.4)	32 (91.4)	85 (82.5)	10 (52.6)	92 (80.0)	3 (42.9)

* Other—Land clearing, wool, among others.

**Table 6:** Cluster characterization according to the prevalence of *Campylobacter* spp., *Salmonella* spp., presumptive ESBL-producing strains of *E. coli*, and presumptive MRSA.

Bacteria		Cluster 1 (n = 49) (“Resistant”)	Cluster 2 (n = 38) (“Campylobacter”)	Cluster 3 (n = 35) (“Salmonella”)	*p*
*E. coli* ESBL	Non detected	81.6 (40)	92.1 (35)	80.0 (28)	0.142
	Detected	18.4 (9)	7.9 (3)	20.0 (7)	
*Salmonella* spp.	Non detected	100 (49)	100 (38)	0 (0)	<0.001
	Detected	0 (0)	0 (0)	100.0 (35)	
*Campylobacter* spp.	Non detected	100 (49)	0 (0)	51.4 (18)	<0.001
	Detected	0 (0)	100 (38)	48.6 (17)	
MRSA	Negative	87.8 (43)	97.4 (37)	100 (35)	0.018
	Presumptive positive	12.2 (6)	2.6 (1)	0 (90)	

**Table 7:** Multinomial logistic regression model to explain the distribution of zoonotic bacteria among small ruminant farms in Portugal’s Central Region.

Clusters	Category		Significance	OR	OR (%)
“Campylobacter” *versus* “Resistant” cluster	Animal species (compared to sheep)	Goat	0.028	0.331	66.9
	District (compared to Viseu)	Castelo Branco	0.007	0.123	87.7
	District (compared to Viseu)	Coimbra	0.163	0.181	81.9
	District (compared to Viseu)	Guarda	0.762	0.835	16.5
	District (compared to Viseu)	Leiria	0.149	0.283	71.7
	Farmer’s practices	-	0.017	0.704	29.6
“Salmonella” *versus* “Resistant” cluster	Animal species (compared to sheep)	Goat	<0.001	0.075	92.5
	District (compared to sheep)	Castelo Branco	0.004	0.060	94.0
	District (compared to sheep)	Coimbra	0.901	0.892	10.8
	District (compared to sheep)	Guarda	0.781	0.833	16.7
	District (compared to sheep)	Leiria	0.294	0.389	61.1
	Farmer’s practices	-	0.60	0.737	26.3

## Data Availability

The original contributions presented in this study are included in the article/Supplementary Materials. Further inquiries can be directed to the corresponding author.

## Supplementary Materials

The following supporting information can be downloaded at: https://www.mdpi.com/article/10.3390/pathogens14111081/s1, Table S1: Questionnaire answers; Table S2: KAP responses.
