# Supplementary material for: Cross-Sectional Study on Zoonotic Bacteria Carriage by Small Ruminants from Portugal’s Central Region
Source: Pathogens. 2025 Oct 23;14(11):1081. doi: 10.3390/pathogens14111081 (PMC12655204; doi:10.3390/pathogens14111081)
Supplement: Supplementary file 1 [file pathogens-14-01081-s001.zip › pathogens-3919511-supplementary.pdf]

Supplementary Table S1: Questionnaire answers

BIOSSECURITY

Is the outer perimeter of the farm fully fenced, preventing the entry of unauthorized people, animals, and vehicles into the farm?

- Yes (1)
- No (0)

Are the access points to the farm kept closed?

- Yes (1)
- No (0)

Is the farm, including the food and bedding material storage areas, fenced off from the entry of pets (e.g., dogs, cats)?

- Yes (1)
- No (0)

Does the farm have a functional wheel wash (vehicle disinfection)?

- Yes (1)
- No (0)

Are the facilities (sheepfold) cleaned and disinfected weekly?

- Yes (1)
- No (0)

Do you use pressure washing equipment for cleaning the farm?

- Yes (1)
- No (0)

Do you use approved disinfectants (listed in the veterinary biocide list) for disinfecting the farm?

- Yes (1)
- No (0)

Do you clean the feeders and drinkers weekly?

- Yes (1)
- No (0)

Do you have pest control (insects and rodents) on your farm?

- Yes (1)
- No (0)

Do the employees change their clothing and footwear upon entering the farm?

- Yes (1)
- No (0)

Do visitors change their clothing and footwear before entering the farm?

- Yes (1)
- No (0)

When animals enter the farm, do you ensure they are screened for other diseases, besides brucellosis?

- Yes (1)
- No (0)

Upon arrival at the farm, are the animals placed in quarantine?

- Yes (1)
- No (0)

Do the animals drink water from the public supply or private sources (well, pond, etc.) that is tested at least once a year?

- Yes (1)
- No (0)

Is the animals' feed stored in a designated and protected space?

- Yes (1)
- No (0)

Are all carcasses collected by the SIRCA or buried according to the regulations/good practices for carcass disposal?

- Yes (1)
- No (0)

Is the manure treated (left in the sun or subjected to composting) before being used as fertilizer or disposed of?

- Yes (1)
- No (0)

GENERAL INFORMATION ON THE USE OF ANTIBIOTICS

Do your animals receive antibiotics (administered by you or the veterinarian)?

- Yes

- No

Do you have antibiotics stored to use on the farm?

- Yes
- No

Please indicate which antibiotics you usually have on your farm:
Mark all applicable options.

- Penicillin + dihydrostreptomycin (e.g., Pendistrep, Sorobiotico)
- Oxytetracycline (e.g., Calimicina, Engemicina, Oxymycin, Tenaline, Terramicina)
- Tilmicosin (e.g., Tilmovet)
- Cephalosporins (e.g., Ceporex)
- Ampicillin and amoxicillin (e.g., Albipen, Clamoxil, Noroclav, Vetrimoxin)
- Tulathromycin (e.g., Tulissin)
- Fluoroquinolones (e.g., Alsir, Enrotron, Marbocyl)
- Florfenicol (e.g., Nuflor, Resflor)
- Lincomycin + Spectinomycin (e.g., Linco-Spectin)
- Amikacin (e.g., Gabrocol)

Please indicate the 3 problems that most frequently lead to the use of antibiotics on your farm:
Mark all that apply.

- Loss of hunger
- Diarrhea
- Parturition’s difficulties (retained placenta, abortion, metritis)
- Respiratory problems
- Mastitis
- Lameness
- Weight loss
- Other

What do you do with the milk from animals treated with antibiotics while the withdrawal period is in effect?

- Discard it into the sewage

- Discard it on the ground
- Give it to the calves/other animals
- Dry off the animals
- Other

During the treatment period of your animals, what do you do with the manure they produce?

- Manure pit
- Land
- Burial
- Incineration
- Other

Have you received training on:

- Use of antibiotics in animals
- Antibiotic resistance
- Antibiotic residues in animal-derived food
- General information about antibiotics
- I have received training on all the above topics
- I have not received any training on the subject

KNOWLEDGE

Antibiotics can be used to:

- Treat viral infections (0)
- Treat bacterial infections (1)
- Treat parasitic infections (0)

Antibiotics have the effect of:

- Anti-inflammatory (0)
- Analgesic and antipyretic (0)
- None of the above (1)

What is the purpose of using antibiotics?

- Growth promoter (0)
- Disease prevention (0)
- Disease treatment (1)

The use of antibiotics in animals:

- Has no side effects, always providing more benefits than risks (0)
- Will not affect me or my family in the future, even indirectly (0)
- Increases the risk of antimicrobial resistance in animals (1)

What do you understand by the withdrawal period of an antibiotic?

- It is the period during which the antibiotic is being administered to the animal (0)
- It is the period after administration, during which the animal or its products (meat/milk) can’t be consumed (1)
- It lasts for one week, regardless of the type of antibiotic (0)

What can delay the development of antimicrobial resistance?

- Reducing the use of antibiotics (1)
- Reducing the duration of treatment (0)
- Reducing the dose administered to the animal (0)

Animal-derived food products (milk, meat, etc.) consumed before the end of the withdrawal period:

- Are safe for animal consumption (e.g., feeding milk to lambs) (0)
- May promote the development of antibiotic resistance in the consumer (1)
- Are safe for the consumer (0)

ATTITUDES

Do you ALWAYS consult the Veterinarian before administering antibiotics to the animals?

- Yes (1)
- No (0)

Do you suspend the antibiotic as soon as the animal improves, even before completing the recommended treatment period?

- Yes (0)
- No (1)

Do you usually modify the antibiotic dose according to the animal's condition?

- Yes (0)
- No (1)

Do the implementation of appropriate biosecurity and management measures allow reducing the use of antibiotics?

- Yes (1)
- No (0)

Do you believe that vaccination helps reduce the use of antibiotics?

- Yes (1)
- No (0)

Can reducing the use of antibiotics in animal production lead to a decrease in antimicrobial resistance?

- Yes (1)
- No (0)

Do you believe that the use of antibiotics in animal production can impact human health?

- Yes (1)
- No (0)

PRACTICES

When you have a sick animal on your farm, how do you usually act?

- I self-medicate, based on my experience (0)
- I call or ask for advice from the Veterinarian (1)
- I wait and see how the animal evolves (0)

How do you acquire antibiotics for your farm?

- I buy them abroad (0)
- I buy them online (0)
- I buy them at a Veterinary Medicine Sales Point or from the Veterinarian (1)

Do you keep a record of antibiotic use in the medication book?

- Yes, always (1)
- Sometimes (0)
- Never (0)

Who administers the antibiotics on your farm?

- Myself (0)

- The Veterinarian (administers all doses or administers the first dose and guides me in administering the subsequent ones) (1)
- Another technician (0)

What do you do with empty antibiotic containers, needles, and syringes?

- Household waste (0)
- Burn them (0)
- Leave them at the pharmacy (1)
- Give them to the Veterinarian (1)

Do you usually use leftover antibiotics to treat other animals that become sick?

- Yes (0)
- Sometimes (0)
- No (1)

Where do you store antibiotics and other medications?

- In the barn (0)
- At my home (0)
- In the medication cabinet (1)
- In the medication refrigerator (1)

SOCIO-DEMOGRAPHIC INFORMATION

What is your gender?

- Female
- Male
- Non-binary

How old are you?

- 16-18 years
- 18-30 years
- 31-40 years
- 41-50 years
- 51-66 years
- > 66

How many years of experience do you have as a sheep/goat farmer?

- < 5
- 6-10
- 11-20
- 21-30
- 31-40
- > 40

What is your academic qualification?

- I did not study
- Primary school (4th grade)
- 6th grade
- 9th grade
- Completed secondary school (12th grade)
- Vocational technical course
- Bachelor's degree
- Master's degree
- Doctorate

District of the farm

- Aveiro
- Castelo Branco
- Coimbra
- Guarda
- Leiria
- Lisboa
- Portalegre
- Santarém
- Viseu

Species produced on your farm (cohabitants)

- Sheep

- Goats
- Mixed (sheep/goat)
- Other

If you selected "Other," please specify

What is your production objective?

- Milk
- Meat
- Meat + Milk
- Other (e.g., land clearing, wool, etc.)

Production system

- Intensive (animals permanently housed)
- Extensive (animals permanently on pasture)
- Semi-extensive (animals spend more time on pasture than in the barn)
- Semi-intensive (animals spend more time in the barn than on pasture)

Type of production

- Conventional production
- Organic production

Supplementary Table S2: KAP responses

KNOWLEDGE	Good knowledge (%)	Poor knowledge (%)
**Antibiotics can be used to**	57.4	42.6
**Antibiotics have the effect of:**	32.8	67.2
**What is the purpose of using antibiotics?**	91.0	9.0
**The use of antibiotics in animals:**	68.0	32.0
**What do you understand by the withdrawal period of an antibiotic?**	82.8	17.2
**What can delay the development of antimicrobial resistance?**	54.1	45.9
**Animal-derived food products (milk, meat, etc.) consumed before the end of the withdrawal period:**	76.2	23.8

ATTITUDES	Good attitudes (%)	Poor attitudes (%)
**Do you ALWAYS consult the Veterinarian before administering antibiotics to the animals?**	86.1	13.9
**Do you suspend the antibiotic as soon as the animal improves, even before completing the recommended treatment period?**	60.6	39.3
**Do you usually modify the antibiotic dose according to the animal's condition?**	85.2	14.8
**Do the implementation of appropriate biosecurity and management measures allow reducing the use of antibiotics?**	92.6	7.4
**Do you believe that vaccination helps reduce the use of antibiotics?**	97.5	2.5
**Can reducing the use of antibiotics in animal production lead to a decrease in antimicrobial resistance?**	95.9	4.1
**Do you believe that the use of antibiotics in animal production can impact human health?**	82.0	18.0
**PRACTICES**	Good practices (%)	Poor practices (%)
**When you have a sick animal on your farm, how do you usually act?**	79.5	20.5
**How do you acquire antibiotics for your farm?**	90.2	9.8
**Do you keep a record of antibiotic use in the medication book?**	37.7	62.3
**Who administers the antibiotics on your farm?**	79.5	20.5
**What do you do with empty antibiotic containers, needles, and syringes?**	53.3	46.7
**Do you usually use leftover antibiotics to treat other animals that become sick?**	50.8	49.2
**Where do you store antibiotics and other medications?**	52.5	47.5
**BIOSSECURITY**	Measure applied (%)	Measure not applied (%)
**Is the outer perimeter of the farm fully fenced, preventing the entry of unauthorized people, animals, and vehicles into the farm?**	71.3	28.7
**Are the access points to the farm closed?**	54.9	45.1
**Is the farm, including the food and bedding material storage areas, fenced off from the entry of pets (e.g., dogs, cats)?**	45.9	54.1
**Does the farm have a functional wheel wash (vehicle disinfection)?**	2.5	97.5
**Are the facilities (sheepfold) cleaned and disinfected weekly?**	9.8	90.2
**Do you use pressure washing equipment for cleaning the farm?**	11.5	88.5

**Do you use approved disinfectants (listed in the veterinary biocide list) for disinfecting the farm?**	50.0	50.0
**Do you clean the feeders and drinkers weekly?**	77.0	23.0
**Do you have pest control (insects and rodents) on your farm?**	22.1	77.9
**Do the employees change their clothing and footwear upon entering the farm?**	33.6	66.4
**Do visitors change their clothing and footwear before entering the farm?**	2.5	97.5
**When animals enter the farm, do you ensure they are screened for other diseases, besides brucellosis?**	77.0	23.0
**Upon arrival at the farm, are the animals placed in quarantine?**	68.0	32.0
**Do the animals drink water from the public supply or private sources (well, pond, etc.) that is tested at least once a year?**	41.8	58.2
**Is the animals' feed stored in a designated and protected space?**	85.2	14.8
**Are all carcasses collected by the SIRCA or buried according to the regulations/good practices for carcass disposal?**	51.6	48.4
**Is the manure treated (left in the sun or subjected to composting) before being used as fertilizer or disposed of?**	54.9	45.1
